# Defining eligible patients for allele-selective chemotherapies targeting NAT2 in colorectal cancer

**DOI:** 10.1038/s41598-020-80288-z

**Published:** 2020-12-31

**Authors:** Veronica Rendo, Snehangshu Kundu, Natallia Rameika, Viktor Ljungström, Richard Svensson, Kimmo Palin, Lauri Aaltonen, Ivaylo Stoimenov, Tobias Sjöblom

**Affiliations:** 1 Science for Life Laboratory, Department of Immunology, Genetics and Pathology, Uppsala University, 751 85 Uppsala, Sweden; 2 Dana-Farber Cancer Institute, 450 Brookline Avenue, Boston, 02215 USA; 3 Uppsala Drug Optimization and Pharmaceutical Profiling Facility (UDOPP), SciLifeLab Chemical Biology Consortium Sweden (CBCS), Department of Pharmacy, Uppsala University, 751 23 Uppsala, Sweden; 4 SciLifeLab Drug Discovery and Development Platform, ADME of Therapeutics facility (UDOPP), Department of Pharmacy, Uppsala University, 751 23 Uppsala, Sweden; 5 Applied Tumor Genomics Research Program, Faculty of Medicine, University of Helsinki, Biomedicum Helsinki, P.O. Box 63, Haartmaninkatu 8, 00014 Helsinki, Finland; 6 Department of Medical and Clinical Genetics, University of Helsinki, Biomedicum Helsinki, P.O. Box 63, Haartmaninkatu 8, 00014 Helsinki, Finland

**Keywords:** Cancer genetics, Colorectal cancer, Cancer metabolism

## Abstract

Therapies targeting somatic bystander genetic events represent a new avenue for cancer treatment. We recently identified a subset of colorectal cancer (CRC) patients who are heterozygous for a wild-type and a low activity allele (NAT2*6) but lack the wild-type allele in their tumors due to loss of heterozygosity (LOH) at 8p22. These tumors were sensitive to treatment with a cytotoxic substrate of NAT2 (6-(4-aminophenyl)-N-(3,4,5-trimethoxyphenyl)pyrazin-2-amine, APA), and pointed to NAT2 loss being a therapeutically exploitable vulnerability of CRC tumors. To better estimate the total number of treatable CRC patients, we here determined whether tumor cells retaining also other NAT2 low activity variants after LOH respond to APA treatment. The prevalent low activity alleles NAT2*5 and NAT2*14, but not NAT2*7, were found to be low metabolizers with high sensitivity to APA. By analysis of two different CRC patient cohorts, we detected heterozygosity for *NAT2* alleles targetable by APA, along with allelic imbalances pointing to LOH, in ~ 24% of tumors. Finally, to haplotype the *NAT2* locus in tumor and patient-matched normal samples in a clinical setting, we develop and demonstrate a long-read sequencing based assay. In total, > 79.000 CRC patients per year fulfil genetic criteria for high sensitivity to a *NAT2* LOH therapy and their eligibility can be assessed by clinical sequencing.

## Introduction

Colorectal cancer (CRC) is the third most common form of cancer in the world with more than 1.4 million newly diagnosed cases and almost 700.000 deaths in 2012^1^. Surgery followed by conventional chemotherapy remains the preferred treatment of CRC. Although several targeted therapies are used in the clinical management of unresectable CRCs^2,3^, the development of novel therapeutic avenues is warranted because of resistance development. Several recent strategies seek to target somatic loss of bystander genes located near tumor suppressor genes for cancer therapy^4–10^. We recently reported that LOH at the *NAT2* locus on 8p22 can be exploited for therapy using APA, a cytotoxic low molecular weight compound^11^. The *NAT2* gene encodes N-acetyltransferase 2, a cytosolic enzyme involved in phase II metabolism of xenobiotics, which is highly polymorphic and has > 100 known single nucleotide variants (SNVs) in the protein coding sequence. The majority of these variants give rise to protein products with lower acetylator activity than the wild-type allele^12^. The expression of NAT2 is largely confined to epithelial cells of the gastrointestinal tract and liver, but NAT2 protein has also been detected in lung, bladder and other tissues exposed to exogenous compounds^13,14^. Due to chromosome arm LOH at 8p22 during the early development of CRC, a patient heterozygous for a rapid and a slow NAT2 allele may lose the rapid variant in the tumor cells, effectively rendering them deficient in NAT2 activity and sensitive to a cytotoxic substrate of NAT2^11^. As the normal epithelial cells of gut and liver express also the wild-type NAT2 allele, they can evade APA toxicity^11^. The number of patients standing to benefit from treatment with APA will depend on (1) the fraction of the CRC patient population that has a targetable NAT2 intermediate acetylator phenotype (i.e. harbor a rapid and a slow NAT2 allele where the slow allele cannot process APA), and (2) the fraction of CRCs that lose their rapid NAT2 allele. It is therefore essential to understand which specific slow NAT2 alleles confer sensitivity to APA and to determine their prevalence in CRC patient populations in order to advance to clinical implementation.

The distribution of *NAT2* alleles differs in populations from geographically separated regions, but combinations of alleles encoding intermediate or low NAT2 enzymatic function are present in all^15^. For example, the wild type allele NAT2*4 and the alleles NAT2*11, NAT2*12, NAT2*13 encode proteins with a rapid acetylator phenotype, whereas the allele groups NAT2*5, NAT2*6, NAT2*7 and NAT2*14 confer the slow acetylator phenotype^16,17^. We focused our previous studies^11^ on tumor cells expressing the wild-type NAT2*4 (rapid) or the NAT2*6 (slow) acetylator allele, and it is currently unknown which, if any, alleles arising from the compendium of NAT2 slow acetylator variants that can be exploited in LOH-based therapy using APA. Once these variants are identified, accurate haplotype resolution at the *NAT2* locus of normal and tumor samples is required for diagnostic purposes. Sanger sequencing and single nucleotide polymorphism (SNP) arrays are limited with respect to haplotype resolution as they rely on statistical inference to resolve complex genotype data^18^. More recently, haplotype phasing of high-throughput short-read next-generation sequencing (NGS) data has been employed, but this method heavily relies on high density of SNPs for accurate inference^19^. As sequencing methods based on single-molecule real-time (SMRT) sequencing can provide more reliable resolution of alleles and haplotypes at a comparable cost per sample^20,21^, we hypothesize that the implementation of these long-read based assays will improve the resolution of *NAT2* haplotypes.

**Figure 1:**
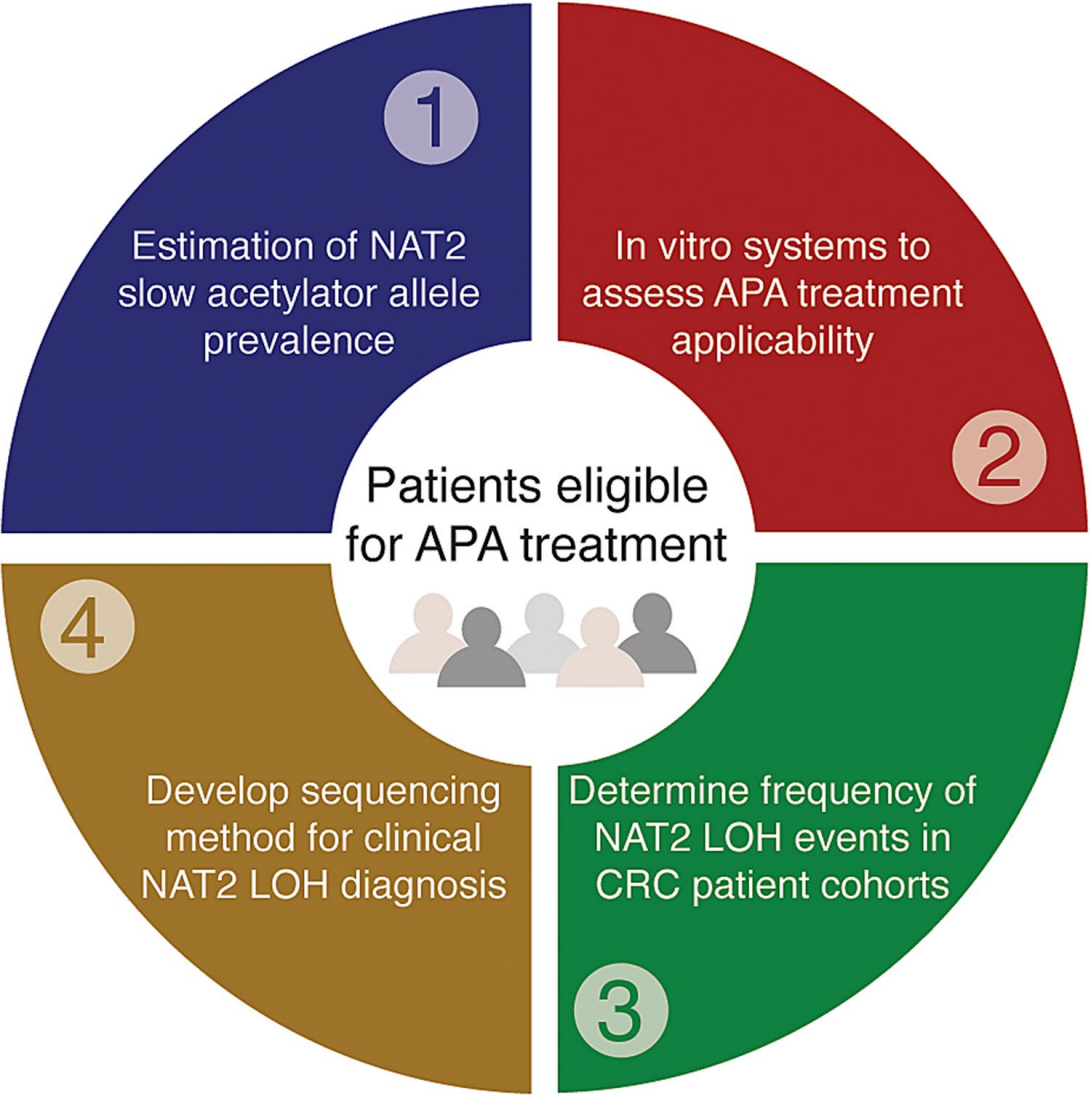
Workflow for the identification of eligible patients for allele-selective chemotherapy with APA.

In the present study, we seek to determine the number of patients who can benefit from a cytotoxic compound (APA) targeting LOH of NAT2 (Fig. 1). First, we construct CRC cell model systems to assess the applicability of APA treatment in cases where the tumor is left with the slow *NAT2* alleles belonging to the groups NAT2*5, NAT2*7 and NAT2*14. Next, we genotype *NAT2* in two independent CRC patient cohorts to determine frequencies of alleles and LOH events. For diagnostic purposes, we finally develop a low-cost long-read sequencing protocol for the multiplexed *NAT2* haplotyping of patient-matched normal and tumor samples.

## Results

### Analysis of NAT2 slow acetylator allele prevalence and estimation of patients eligible for allele selective therapy

We determined the *NAT2* allele group frequencies from the phased haplotypes of 2054 individuals sequenced by the 1000 Genomes Project^22^ and observed that the slow acetylator alleles from the groups NAT2*5, NAT2*6, NAT2*7 and NAT2*14 represented 29.3%, 26.5%, 7.7% and 2.8% of the total alleles, respectively. The fraction of heterozygous individuals with the slow acetylator variants *5 (rs1801280), *6 (rs1799931), *7 (rs72554617) and *14 (rs1801279) were 35.7%, 36.7%, 13.0% and 5.0%, respectively. However, only heterozygous individuals with one slow and one rapid acetylator allele (NAT2*4 or NAT2*12) are considered for allele-selective therapy. Heterozygosity was present in 15.1% (NAT2*5/Rapid), 13.4% (NAT2*6/Rapid), 5.7% (NAT2*7/Rapid) and 1.6% (NAT2*14/Rapid) of samples. We detected only one allele from the group NAT2*10 and no other slow acetylator alleles in the dataset. The data suggest that ~ 35.8% of the global population has an intermediate NAT2 acetylator phenotype through heterozygosity of prevalent rapid and slow *NAT2* alleles. Given that ~ 21% of colorectal tumors undergo LOH at 8p22^23^, we estimate that ~ 79,000 individuals heterozygous for a *NAT2* slow allele could potentially be eligible for therapy every year (given an annual incidence of 1.3 million CRC cases) (Supplementary Table 1). We previously demonstrated that cells encoding the NAT2*6 variant are more sensitive to APA treatment than those expressing a NAT2*4 rapid acetylator allele^11^. The observation that there are almost twice as many intermediate acetylators carrying a slow allele from the groups NAT2*5, NAT2*7 and NAT2*14 as compared to NAT2*6 motivated further functional studies to determine the response of these alternative slow acetylator alleles to APA.

### Prediction of NAT2 haplotypes and LOH events in two independent CRC patient cohorts

**Figure 2:**
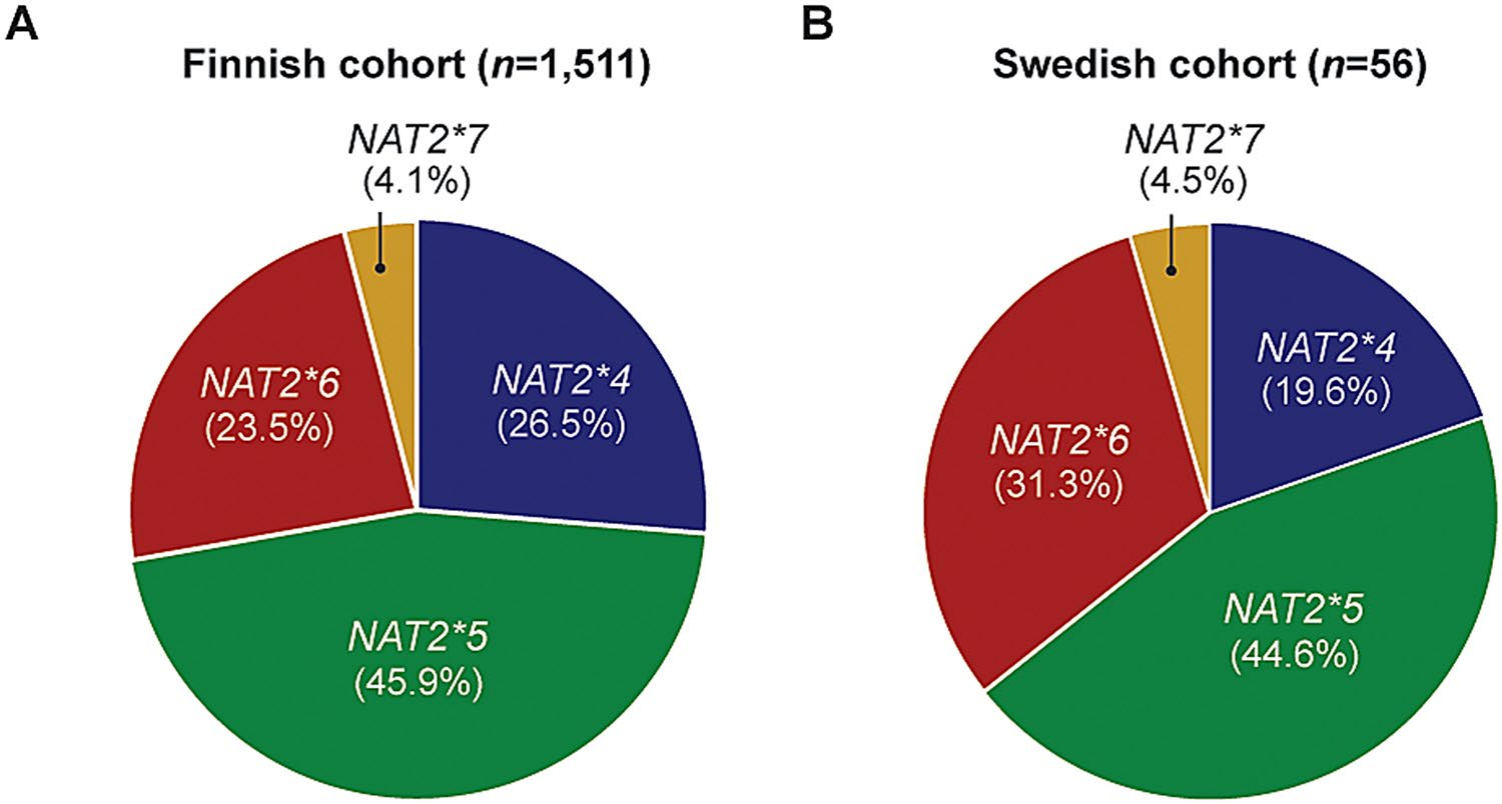
Distribution of *NAT2* allele groups in two colorectal cancer cohorts. Genotyping of tumor and patient-matched normal samples identified three *NAT2* allele groups encoding slow acetylator variants present in a (**A**) Finnish cohort of 1511 patients analyzed by SNP array genotyping and a (**B**) Swedish cohort of 56 patients analyzed by NGS sequencing. The allele NAT2*12 was detected in the cohort of Finnish colorectal cancers, but was excluded as its frequency was < 0.05. Percentages are calculated based on the total number of genotypes obtained for each patient group.

We next sought to identify the frequencies at which the different NAT2 slow variants are present in two independent cohorts of CRC patients. We first genotyped a Finnish cohort of 1611 CRC patients^24^ by querying 7 common allele-classifying *NAT2* SNPs and 11 additional variants in tumor and patient-matched normal samples. We were able to derive the haplotypes of 1,511 individuals, identifying the allele group NAT2*5 (45.9%) as the most frequent followed by *4 (26.5%), *6 (23.5%), *7 (4.1%) and *12 (< 0.1%) (Fig. 2A). Heterozygous individuals carrying a wild-type NAT2*4 allele and a slow acetylator *5A, *5B, *5C, *6A or *7B allele constituted 37.3% of the CRC patients. Next, we determined the fraction of tumors retaining a slow NAT2 acetylator allele after undergoing LOH in chromosome 8p22. Allelic imbalance was observed in 30.9% of all predicted intermediate acetylators, and the wild-type allele was lost in 52.9% of the putative LOH events (Table 1). Considering all tumors, the LOH frequency at the *NAT2* locus in all tumors was 24.3%. We then studied a cohort of 56 Swedish patients by analysis of tumor and patient-matched normal samples whole genome sequenced at a read depth of 90 × . By calling 14 SNP positions in the *NAT2* locus, we were able to define tumor and normal haplotypes in each patient and identify allele losses occurring in the former. The highest allele frequency was present in groups NAT2*5 (44.6%) and *6 (31.3%), followed by NAT2*4 (19.6%) and NAT2*7 (4.5%) (Fig. 2B). For two patients, haplotypes could not be assigned due to a low number of sequencing reads and uncertain base calling. One-quarter (26%) of patients were heterozygous intermediate acetylators carrying NAT2*4/6A or *4/5B, and 3 additional intermediate acetylator individuals (5%) had *4/5A, *4/5C or *4/7B genotypes. Again, LOH events were detected in 19.6% of the total cohort and particularly in 40% of heterozygous patients with the NAT2*4/*5B haplotype. Analyses of these two cohorts revealed five different *NAT2* genotypes in heterozygous patients losing wild-type NAT2 in their tumors (Table 1) and motivated further understanding on whether CRC patients with the NAT2*4/5B or *4/7B haplotypes are also eligible for LOH-based therapy. We did not detect any individual heterozygous for the NAT2*14 slow acetylator allele but included this variant in downstream analyses as tumors retaining the slow allele may also show susceptibility to NAT2 allele-selective compounds.

**Table 1:** Distribution of *NAT2* genotypes and LOH events identified in tumor/normal colorectal cancer pairs in (1) Finnish cohort, (2) Swedish cohort and (3) combined data.

*NAT2* haplotype	Number of individuals			LOH events in intermediate acetylators			Cases where the *4 allele is lost		
	(1)	(2)	(3)	(1)	(2)	(3)	(1)	(2)	(3)
**Rapid acetylators**									
NAT2*4/*4	117	2	119	1	0	1	1	0	1
NAT2*4/*12A	2	0	2	2	0	2	1	0	1
Total	119	2	**121**	3	0	**3**	2	0	**2**
**Intermediate acetylators**									
NAT2*4/*5A	20	1	21	4	0	4	2	0	2
NAT2*4/*5B	328	10	338	95	4	99	50	0	50
NAT2*4/*5C	16	1	17	6	1	7	3	1	4
NAT2*4/*6A	170	5	175	58	0	58	29	0	29
NAT2*4/*7B	30	1	31	11	0	11	8	0	8
Total	564	18	**582**	174	5	**179**	92	1	**93**
**Slow acetylators**									
NAT2*5A/*5A	1	0	1	0	0	0	0	0	0
NAT2*5B/*5B	228	6	234	12	0	12	0	0	0
NAT2*5C/*5C	3	0	3	0	0	0	0	0	0
NAT2*5A/*5B	33	0	33	15	0	15	0	0	0
NAT2*5A/*5C	6	0	6	3	0	3	0	0	0
NAT2*5B/*5C	35	3	38	16	0	16	0	0	0
NAT2*5A/*6A	20	1	21	6	0	6	0	0	0
NAT2*5B/*6A	308	18	326	106	0	106	0	0	0
NAT2*5C/*6A	19	0	19	5	0	5	0	0	0
NAT2*5A/*7B	5	0	5	2	0	2	0	0	0
NAT2*5B/*7B	58	3	61	15	0	15	0	0	0
NAT2*5C/*7B	1	1	1	1	0	1	0	0	0
NAT2*6A/*6A	84	4	88	0	0	0	0	0	0
NAT2*6A/*7B	25	1	26	9	0	9	0	0	0
NAT2*7B/*7B	2	0	2	0	0	0	0	0	0
Total	828	36	**864**	190	0	**190**	0	0	**0**
Total patients	1511	56	**1567**	367	5	**372**	0	0	**95**

NAT2 acetylator phenotype was called based on rs1801279, rs1041983, rs1801280, rs1799929, rs144176822, rs1799930, rs1208, rs1799931, rs2552, rs4646247, rs971473, rs45547533, rs721398 and rs721399 genotypes.

Terms in bold refer to the acetylator phenotype of each tumor (rapid, intermediate or slow) given its NAT2 haplotype.

### Generation of CRC cell model systems expressing NAT2 slow acetylator variants

**Figure 3:**
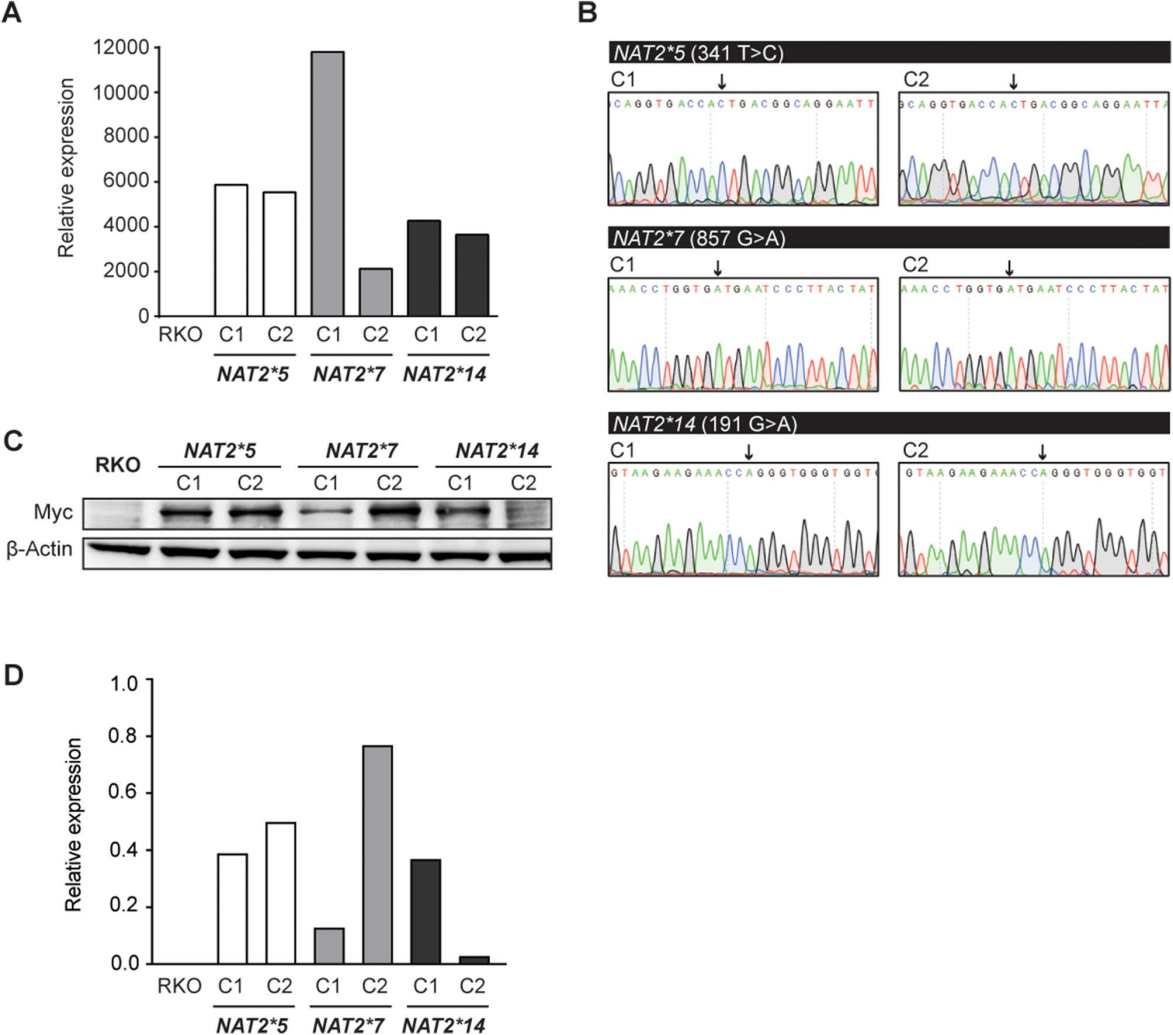
Generation of stable CRC cell clones expressing NAT2 slow acetylator variants. (**A**) Two clones encoding each of the indicated NAT2 slow acetylator variants were chosen for downstream analyses based on their expression of NAT2 RNA relative to parental RKO cells. Data from one representative experiment shown. (**B**) Sanger sequencing revealed the correct 341 T > C, 857 G > A, and 191G > A substitutions in the *NAT2* coding region (black arrows) to generate the NAT2*5, *7 and *14 alleles. (**C**) Protein expression of NAT2 slow acetylator variants by immunoblotting. A Myc-tag antibody was used to detect recombinant NAT2 with β-actin as loading control. Full-length immunoblots are presented in Supplementary Fig. 4. (**D**) Quantification of immunoblots shown in (**C**). Protein expression levels were normalized against β-actin.

To determine which of the common slow acetylator NAT2 alleles can be exploited for NAT LOH therapies, we developed cell model systems expressing the slow acetylator variants encoded by the NAT2*5, *7 and *14 allele groups. The CRC cell line RKO was selected as a genetic background due to its lack of endogenous *NAT2* expression. A total of 6 stable clones encoding NAT2*5, 5 clones encoding *7 and 4 clones encoding *14 were obtained after transduction and selection of RKO cells (Supplementary Fig. 1). Further RT-qPCR analysis allowed selection of 2 clones with similar mRNA expression levels for each NAT2 slow acetylator variant (Fig. 3A). For the final set of 6 clones, the correct genotype and gene expression of the variants rs1801280 (NAT2*5), rs1799931 (NAT2*7) and rs1801279 (NAT2*14) was confirmed by PCR-coupled Sanger sequencing (Fig. 3B) and immunoblotting (Fig. 3C, D). All 3 slow acetylator variant clones grew at similar rates to parental RKO cells when cultured under normal medium conditions (Supplementary Fig. 2). Taken together, the generated cell systems were considered suitable tools for determination of NAT2 kinetic parameters and sensitivity towards agents targeting LOH.

### Quantification of NAT2 catalytic activity in slow acetylator variants

**Figure 4:**
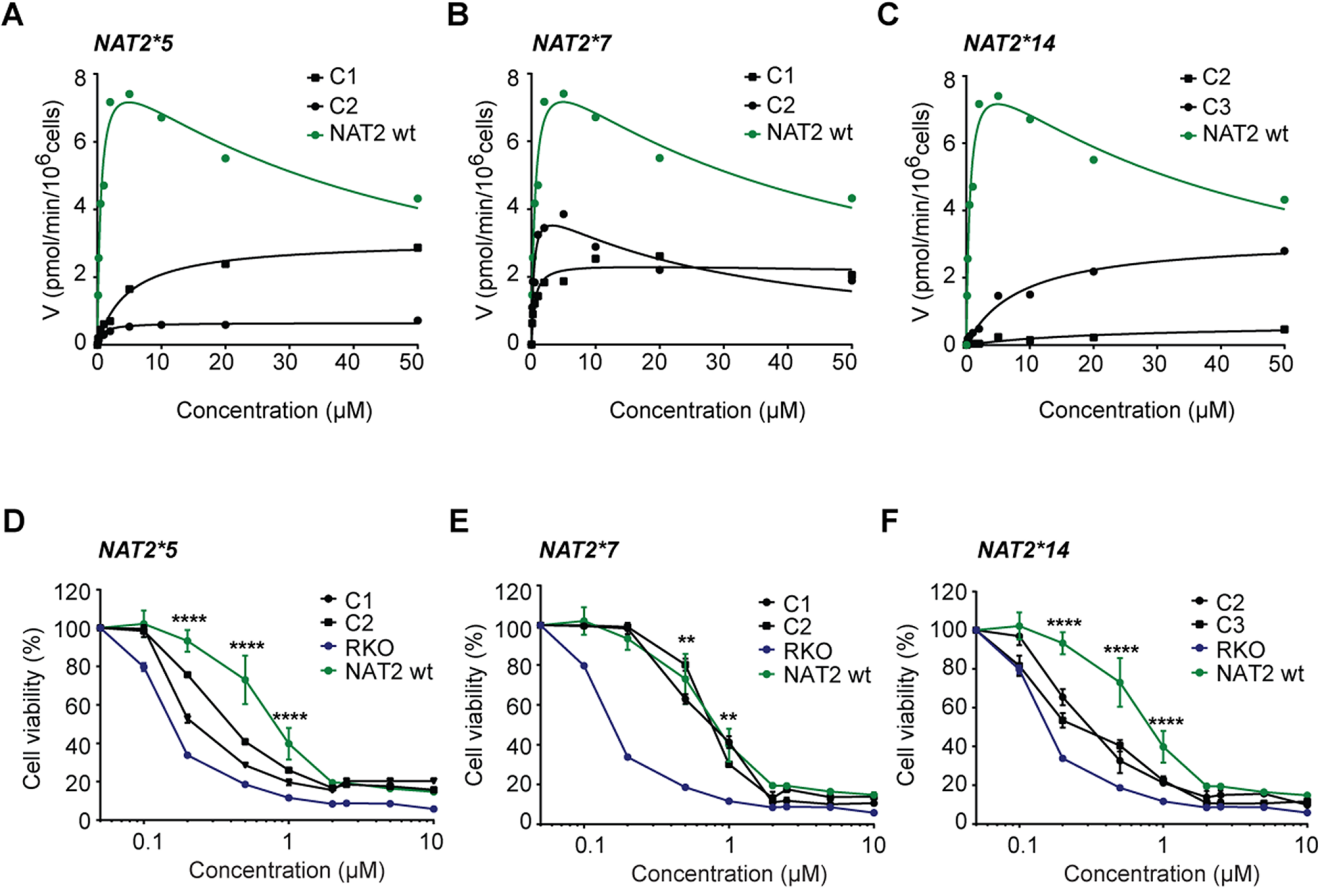
Colorectal cancer cells expressing NAT2_5 and NAT2_14 but not NAT2_7 are sensitive to APA treatment. (**A**–**C**) Quantification of NAT2 catalytic activity in RKO clones expressing (**A**) NAT2_5, (**B**) NAT2_7 and (**C**) NAT2_14 enzymatic variants. The velocity at which APA becomes acetylated was measured by LC–MS/MS at different concentrations. A representative experiment is shown for each enzymatic variant. (**D**–**F**) Dose response for APA in RKO clones expressing the (**D**) NAT2_5, (**E**) NAT2_7 and (**F**) NAT2_14 enzymatic variants. Cell viability was measured by a MTT assay after 72 h. The mean and S.D. of three independent experiments is shown. Data were analyzed using a two-way ANOVA. **p < 0.01 and ****p < 0.0001.

To determine the kinetic parameters underlying acetylation of the NAT2_5, NAT2_7 and NAT2_14 protein variants, cells were treated with increasing concentrations of the cytotoxic NAT2 substrate APA, a substrate for which a 40-fold increased affinity was observed in rapid NAT2_4 versus slow NAT2_6 CRC cells^11^. Impaired selectivity towards APA was observed in NAT2_5 (V_max_/K_m_ = 0.80 and 0.64 µl/min/10^6^ cells) and NAT2_14 variants (V_max_/K_m_ = 0.03 and 0.38 µl/min/10^6^ cells), leading to > 20 and > 100-fold less efficient NAPA formation as compared to the wild-type NAT2 clone (V_max_/K_m_ = 15.5 µl/min/10^6^ cells) (Fig. 4A, C). Interestingly, clones expressing the NAT2_7 variant (V_max_/K_m_ = 11.8 and 5.0 µl/min/10^6^ cells) showed much higher acetylation efficiency, comparable to the wild-type enzyme and one clone also displaying substrate inhibition kinetics (Fig. 4B). The low levels of NAT2 catalytic activity observed in the NAT2_5 and NAT2_14 variants are in agreement with the kinetic profile obtained for NAT2_6-mediated APA acetylation^11^ and suggest that tumor cells retaining these variants after undergoing LOH would have comparable sensitivity levels towards APA treatment.

### Sensitivity of NAT2 slow acetylator variants towards APA treatment

The low molecular weight compound APA shows selective toxicity towards cells expressing the slow acetylator NAT2_6 protein. To investigate whether this sensitivity extends to the remaining NAT2 slow acetylator variants, a full dose–response for APA was determined in cells expressing NAT2_5, -_7 and -_14. Cells expressing the NAT2_5 (IC_50_ = 0.86 µM) and NAT2_14 (IC_50_ = 0.15 µM) slow acetylator variants showed a > fourfold decrease in viability when compared to wild-type NAT2 (Fig. 4D, F). In contrast, NAT2_7-expressing clones had cell viability rates (IC_50_ = 3.46 µM) comparable to wild-type cells (Fig. 4E). To confirm that the observed APA response was dependent on NAT2, we performed a similar dose–response curve with fluorouracil (5-FU), a standard CRC chemotherapy drug which is not metabolized by NAT2. The cell viability of all clones was equally impaired by 5-FU in all assessed NAT2 genotypes (Supplementary Fig. 3). Taken together, we conclude that NAT2*5 and NAT2*14, but not NAT2*7 slow acetylator alleles, encode variants sensitive to APA treatment.

### NAT2 haplotype resolution by multiplex SMRT sequencing

**Figure 5:**
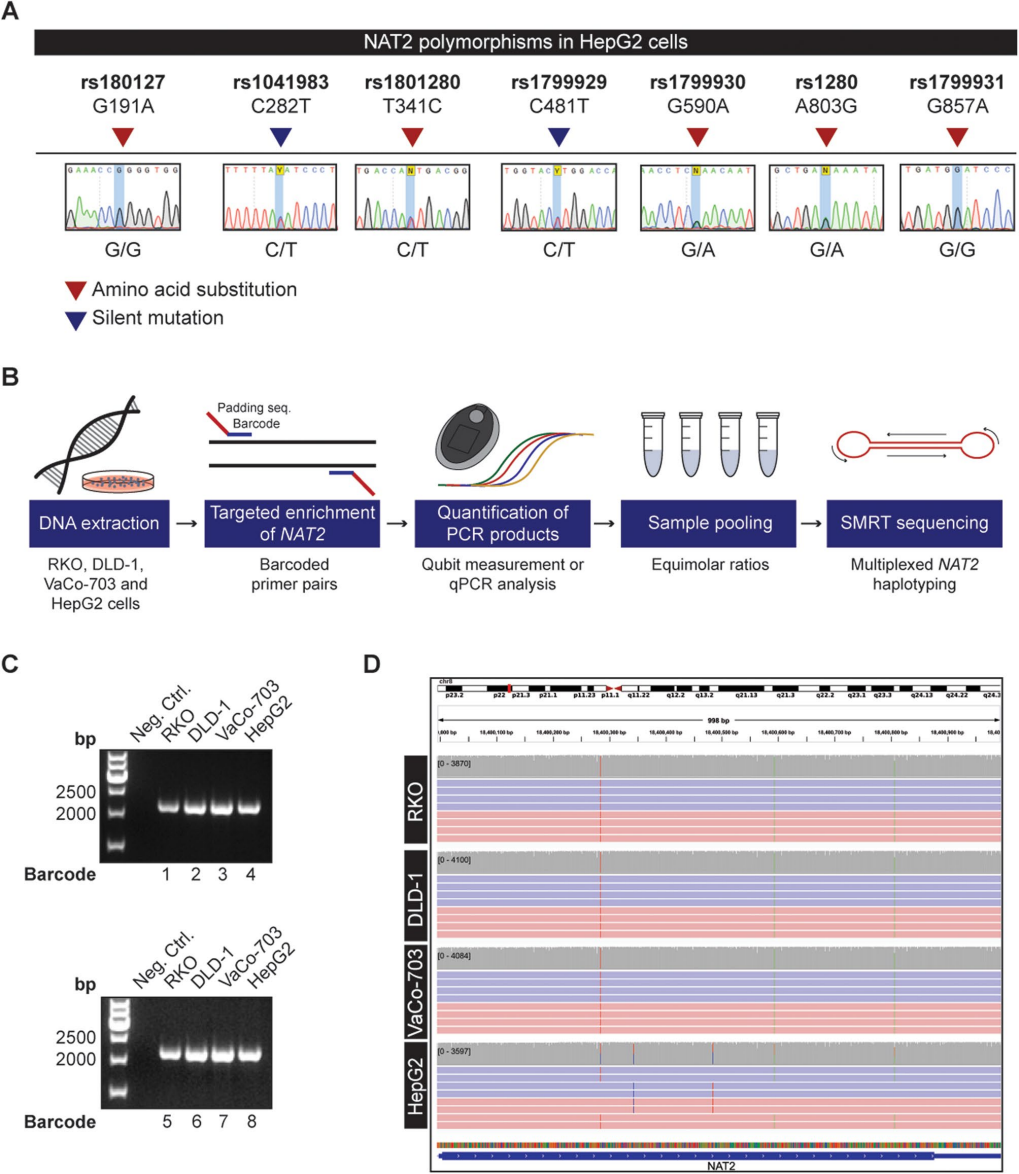
*NAT2* haplotype resolution by multiplex SMRT sequencing. (**A**) Sanger sequencing does not allow the resolution of *NAT2* haplotypes in samples that are polymorphic in more than one position. The genotype of HepG2 cells is shown for each of the seven standard positions used for *NAT2* allele calling. (**B**) Workflow for multiplexed SMRT sequencing. Genomic DNA was extracted from the cancer cell lines to be haplotyped. The *NAT2* region is amplified in each sample by using primer pairs with a unique barcode and a padding sequence. The resulting PCR products were quantified by Qubit measurements or qPCR analysis and pooled in equimolar ratios for multiplexed SMRT sequencing. (**C**) Barcoded PCR products resulting from *NAT2* amplification in RKO, DLD-1, VaCo-703 and HepG2 cell lines with two different sets of primers. Samples were run in a 1.5% agarose gel and water was used as a negative PCR control. Full-length gels are presented in Supplementary Fig. 4. (**D**) Visualization of the individual alleles present in RKO, DLD-1, VaCo-703 and HepG2 cell lines in Integrative Genomics Viewer. De-multiplexed SMRT sequencing reads from each cell line are aligned to the *NAT2* reference sequence. Selection of reads in both positive (red) and negative (blue) direction supporting the NAT2*6A allele in RKO, DLD1, VaCo-703, HepG2 and NAT2*5B allele in HepG2 are shown. The gray stacks above each alignment represent the sequencing coverage. Vertical colored lines illustrate the positions of variation from the reference.

The resolution of NAT2 haplotypes is critical for understanding whether a colorectal tumor has undergone LOH and is eligible for APA treatment. However, conventional Sanger sequencing as well as short read sequencing methods are not able to discern whether polymorphisms are present in the same allele or in different alleles of the *NAT2* gene, thereby impairing the understanding of which specific allele has been lost in tumor cells. Indeed, Sanger sequencing was able to resolve the polymorphisms in the CRC cell lines RKO, DLD-1, and VaCo-703 which are homozygous for all *NAT2* polymorphisms, but not in HepG2 cells where heterozygosity was observed at all 7 polymorphic sites (Fig. 5A). To create a clinically useful multiplexed method for *NAT2* haplotyping, we turned to long-read SMRT sequencing technology, which assigns each *NAT2* polymorphism to a particular DNA strand and allows for the complete resolution of allele groups in a sample. Targeted enrichment of *NAT2* was performed in several samples at the same time, allowing for the quantification and pooling of the resulting PCR products for multiplexed SMRT sequencing (Fig. 5B). First, a 2096 bp long-range PCR product encompassing the entire protein-encoding sequence (exon 2) of *NAT2* was amplified from genomic DNA of RKO, DLD-1, VaCo-703 and HepG2 cell lines and analyzed by PacBio sequencing. Two independent and separately labeled PCR products were obtained for each of the cell lines to assess two alternative sample-pooling strategies. The samples were amplified with barcoded primers with similar amplification efficiency (Fig. 5C; Supplementary Table 2). Four amplicons (one for each cell line) were mixed in equimolar ratio (Pool A) and the other four pooled according to a concentration estimate based on qPCR data (Pool B). The pools were mixed at equimolar ratio and subjected to multiplex sequencing in a single SMRT cell. For the 8 amplicons, we obtained 53,828 reads of the insert with a mean length of 1715 nt and mean quality of 97.32% following 10 sequence passes on average. We based the analysis on sequencing reads with lengths 1500 nt to 2300 nt where the barcode was successfully identified, resulting in 33,817 reads (62.8% of total). Following barcode trimming and read alignment, 99.8% of the sequencing reads aligned to the *NAT2* locus. For the samples from Pool A, the number of aligned reads was 3913 ± 235 (mean and SD), compared to 4540 ± 749 for Pool B. While the amplicon mean coverage was comparable, a larger standard deviation was observed in Pool B. While RKO, DLD-1 and VaCo-703 cells were homozygous in the sequenced region and carried the NAT2*6A allele as suggested by Sanger sequencing, HepG2 cells were polymorphic in 7 sites and the haplotype calling from long-read sequencing revealed the *NAT2* slow-acetylator alleles NAT2*6A and NAT2*5B (Fig. 5D). Taken together, these results demonstrate SMRT sequencing as a valid haplotyping tool to predict NAT2 acetylator phenotypes in cancer patients and their tumors.

### NAT2 acetylator phenotype prediction by SMRT sequencing of tumor-normal pairs

After confirming that NAT2 haplotype resolution is feasible by SMRT sequencing, we aimed to resolve the genotype of 74 CRC tumor and patient-matched normal tissues, which included the Swedish cohort. We sequenced the barcoded *NAT2* amplicons from the normal tissues in two separate SMRT cells with 37 samples each, whereas the patient-matched tumor samples were sequenced in three additional SMRT cells with ~ 25 samples each, aiming for better tumor coverage. Following removal of short sequencing reads (< 1500 nt), barcode de-multiplexing and read alignment we observed that mean *NAT2* coverage per sample was 1547 × in the normal tissues and 2229 × for the tumor samples, which was deemed sufficient to call the haplotypes. We called the haplotypes from the reads covering all 7 allele-classifying polymorphic sites (rs1801279, rs1041983, rs1801280, rs1799929, rs1799930, rs1208, rs1799931) (Supplementary Table 3). Sample N_ID:128 was covered by 175 reads, from which 147 reads span all 7 polymorphic sites and was called as homozygous for the NAT2*5B allele. In the corresponding tumor sample (T_ID:127) the increase of coverage to 2235 × did not reveal a second allele. In sample N_ID:148 with similarly low coverage (238 ×), two alleles were resolved (NAT2*5B/*6A). The allele groups NAT2*4, *5, *6 and *7 were detected in 22.3%, 50.7%, 20.9% and 6.1% of patients, respectively. The data from the complete *NAT2* haplotypes implied that 25 individuals (33.8%) were intermediate acetylators, consistent with the genotype and imputed haplotype data from the SNP arrays and NGS sequenced cohorts. We detected LOH of NAT2 in 19 tumor samples (25.7%) of which 13 were from individuals with an intermediate acetylator phenotype. The LOH events in the intermediate acetylators indicated that the wild-type allele was lost in 3 cases (4% of total). The long-read sequencing approach additionally identified a novel wild-type NAT2*4 allele with a rare synonymous SNP (rs144176822). We conclude that SMRT sequencing enables NAT2 haplotype resolution for multiple samples by analyzing sequencing reads spanning the *NAT2* gene.

## Discussion

The vast majority of human cancers exhibit genomic instability at the chromosomal level. Such cancers acquire somatic changes in chromosomal number and structure throughout tumor progression^25,26^. Cancer vulnerabilities emerging from these genomic losses have been proposed as promising putative targets for the selective treatment of solid tumors. Examples include the loss of bystander metabolic enzymes lost in conjunction with tumor suppressor genes, enabling inhibition by collateral lethality^4–7^. Similarly, loss of essential genes in the spliceosome or proteasome can render cancer cells sensitive to further suppression of these genes^8–10^. In a previous study, we have focused on LOH events reducing the genetic complexity of tumors and have demonstrated that NAT2 could serve as a target in colorectal cancer^11^. We focused on NAT2*6 heterozygous individuals which lose the functional *NAT2* allele in their tumors (as *6 is the slow acetylator allele with the highest heterozygous population frequency) and demonstrated that cell lines and tumors expressing the slow acetylator NAT2*6 allele are more sensitive to APA treatment than those expressing a rapid acetylator NAT2 allele. Based on structural similarity with kinase inhibitors, we additionally performed a kinome screen to profile binding of APA and its acetylated product NAPA with respect to > 300 kinases, and identified differential binding to Aurora Kinase A (AURKA) which is a known drug target in cancer. We were able to determine binding of APA to AURKA, activation of phospho-AURKA in slow but not rapid acetylator tumor cells, and mitotic arrest along with formation of multipolar mitotic spindles in CRC cells expressing the slow acetylator variant. In this study, we hypothesized that individuals carrying other slow variant alleles may also be eligible for APA treatment. Tumor cells encoding the slow acetylator variants NAT2*5 and *14 indeed responded to APA treatment and had similar kinetic profiles when compared to cells expressing the *6 allele group. This is in concordance with previous analyses of sulfamethazine N-acetylation in human hepatocytes, where NAT2-mediated substrate processing occurs at similar rates for all slow acetylator variants^27^. In contrast, cells encoding the NAT2*7 variant showed higher efficiency of APA acetylation and responses to APA treatment that are comparable to cells expressing wild-type NAT2 activity. We hypothesize that this effect is particular for APA.

Haplotyping of *NAT2* in two different colorectal cancer cohorts revealed several combinations of alleles that encode intermediate acetylator phenotypes. For patients heterozygous for *NAT2*, we observed an almost equal probability of losing either allele after the tumor undergoes LOH. The LOH frequency for the *NAT2* locus was > 23% in both cohorts, which is considerably higher than previously reported^28^. This discrepancy could be a result of population specific factors, a difference in the number of patients and most importantly of the amount of SNP variation probed. Besides detecting a loss of the wild type NAT2*4 allele in tumors from NAT2*4/6A individuals (17%), we observed that the same allele can be lost in the tumors of NAT2*4/*5B (15%) and NAT2*4/*7B (27%) individuals. The largest number of intermediate acetylators in both patient cohorts carried a NAT2*4/*5B genotype and, despite the lower LOH frequency observed in the NAT2*4/*5B genetic background compared to the LOH frequency in NAT2*4/6A or NAT2*4/*7B backgrounds, the NAT2*4/*5B intermediate acetylators contributed with the most cases of potentially eligible patients (3.3% of total haplotyped individuals). All intermediate acetylators in our cohorts which lost a wild-type allele in the tumor, account for ~ 6% of cases. These results significantly expand the population of patients eligible for LOH therapy and motivate the development of practical diagnostic tools to identify this clinically relevant subset. No carriers of the NAT2*14 slow acetylator allele were identified, presumably because this allele is not present in the studied populations^15,29^. As NAT2*14A or NAT2*14B haplotypes are found in Spanish (0.39%), German (0.13%) and US Caucasian (0.12%) populations, eligible patients could be found in these cohorts^15^. Despite their low frequency, our functional data suggests that tumors encoding the NAT2*14 variant are equally sensitive to APA treatment and should be considered for LOH-base therapy.

Routine identification of NAT2 intermediate acetylators in the clinic requires a practical genotyping tool, able to resolve the haplotypes with high accuracy and allowing for multiplexing. Commonly used methods such as Sanger sequencing and SNP arrays employ statistical inference to resolve haplotypes, which can lead to an ambiguous interpretation of the data^18^. Alternatively, haplotype resolution can be performed from short reads obtained from next-generation sequencing, but the output is highly dependent on the frequency of the polymorphism that needs to be inferred. We developed SMRT sequencing of NAT2 as a practical diagnostic technology, assigning the genetic variation found in the *NAT2* locus to each DNA strand of the individual and potentially allowing the identification of LOH events in the patient’s tumor. By using SMRT sequencing, we were able to resolve the different *NAT2* alleles present in several cell lines and developed a pipeline for multiplexed analysis. We resolved both *NAT2* alleles (N_ID:148, Supplementary Table 2) from as little as 181 sequencing reads spanning the protein-coding part of the gene and observed on average 43,208 such alleles per SMRT cell. This suggests that one SMRT cell can be used to resolve the *NAT2* haplotypes of more than 200 samples, provided equimolar DNA pooling and barcode availability. This method can now be used as a tool for haplotyping colorectal cancers and patient-matched normal samples, with the aim of identifying *NAT2* heterozygous individuals losing the functional NAT2 alleles in their tumors. As NAT2 is involved in the metabolism of clinically used drugs^30^, we envision that this test may complement routine NAT2 pharmacogenetic analyses.

To estimate LOH frequency from patient-matched tumor-normal samples we used three different methods: SNP-array genotyping, whole-genome sequencing and long-read SMRT sequencing. Genomic arrays and next-generation sequencing approaches allow for the detection of modest allelic imbalances in tumor samples, and provide increased resolution capacity for highly polymorphic loci when compared to LOH blots. However, it’s important to note that no sequencing method on its own can report an LOH event with absolute confidence. The major factors for this uncertainty are: (1) variable tumor purity, (2) tumor heterogeneity and clonality of LOH events, and (3) potential allele biases in the library preparation, sequencing and analysis of the samples. From the three implemented methods, the long-read SMRT sequencing can be considered the best haplotyping strategy since the evidence for each allele is direct and based on independently sequenced single molecules covering both directions of the DNA strands. This is clearly demonstrated by the detection of the rare g.18400582G > T polymorphism and its unambiguous assignment to a novel NAT2*4 allele in a NAT2*4/5B genotype context. The drawback of long-read sequencing is in the sample preparation steps, which may be more laborious than SNP array genotyping or whole-genome sequencing. Long-read SMRT sequencing has been previously proposed for haplotyping of highly polymorphic loci^20,21^, and here we suggest for the first time NAT2 LOH detection as an application. The LOH detection protocol based on long-read sequencing needs further refinement before being accepted as a diagnostic grade protocol, especially given the ambiguity of the allelic imbalance threshold above which one should call an LOH event. However, the long-read sequencing can easily be applied in most clinical laboratories as a low-cost multiplexing method for simultaneous haplotyping of tumor and normal samples, with a possible LOH suggestion in the tumor.

Given the LOH frequencies detected in our studied cohorts, and the response of the different NAT2 slow acetylator variants to APA, we estimate that each year at least 79.000 worldwide CRC patients could benefit from LOH-based targeting. However, the NAT2-allele selective therapy may have broader cancer applicability. In addition to the expression of NAT2 in the tissues of the gastrointestinal tract and liver, detectable protein levels are present in the lung, bladder, ureter and kidneys^13,14^. Cancers of the liver, lung and kidney are also prone to LOH in the *NAT2* locus on chromosome 8p22^23^, in similar frequency to that of colorectal cancer (Supplementary Table 1). Taken together, these results suggest that NAT2-targeted therapy can be beneficial to a larger number of cancer patients.

## Methods

### Ethical permits

Sequence analyses of patient samples were performed under ethical permits from EPN Uppsala (2007:116) and the Ethics Committee of the Hospital District of Helsinki and Uusimaa following participant informed consent. Signed informed consent or authorization from the National Supervisory Authority for Welfare and Health has been obtained for all Finnish sample materials used. All analyses were performed in accordance with relevant guidelines and regulations.

### Generation of stable clones with ectopic expression of slow NAT2 variants

The human colorectal cancer cell line RKO (ATCC) was maintained in McCoy’s 5A medium supplemented with 10% Fetal Bovine Serum and 1% Pen Strep (Life Technologies). Lentiviral particles encoding NAT2*5, NAT2*7 and NAT2*14 slow acetylator variants within the Lv125C backbone sequence were acquired from LabOmics. The day before transduction, 50,000 cells were plated in a 24-well plate. Viruses were diluted in 250 µL of normal growth medium containing 7.5 mg/mL sequa-brene (Sigma) and added to each well. After 24 h of incubation at 37 °C, the virus-containing medium was replaced with fresh normal growth medium. After 48 h, transduced cells were selected with 1 µg/mL puromycin (Thermo Fisher Scientific) for 10 days. Next, 3 cell pools of each NAT2 slow acetylator variant were subjected to single clone selection and screening. To confirm the genotype of each clone, genomic DNA was isolated following a standard protocol for cultured cells (Macherey–Nagel) and amplification of the lentiviral *NAT2* insert was performed by PCR with the forward primer 5′-GCGGTAGGCGTGTACGGT-3′ and reverse primer 5′-ATTGTGGATGAATACTGCC-3′. Each reaction contained 1 × Phusion HF Buffer (Thermo Scientific), 0.2 mM dNTPs, 0.2 µM forward and reverse primers, 0.02 U/µL Phusion DNA Polymerase (Thermo Scientific) and 6 ng of genomic DNA. The PCR was performed in a 2720 Thermal Cycler (Applied Biosystems) and consisted of 1 cycle of 98 °C for 30 s and 30 cycles of 98 °C for 10 s, 61 °C for 15 s and 72 °C for 20 s followed by 1 cycle of 72 °C for 10 min. Next, reactions containing 20 ng of PCR product and 4 pmol of the corresponding primer were prepared for Sanger sequencing on an ABI 360xl instrument (Applied Biosystems). Sequence traces were analyzed in SnapGene Viewer.

The rapid NAT2*4 acetylator variant was generated in a previous study^11^ by transfection of a pCMV6-Entry-NAT2 mammalian expression vector containing a Myc-tagged NAT2 open reading frame. To obtain a rapid acetylator phenotype, site directed mutagenesis of rs1799930 (A590G) was performed using the QuickChange II Site-Directed Mutagenesis Kit (Agilent Technologies) in 50 μL reactions containing 1 × reaction buffer, 125 ng of each primer, 0.2 mM dNTPs and 20 ng of dsDNA template. Primers used for site-directed mutagenesis included forward 5′-GAAGAGGTTGAAGAAGTGCTGAGAAATATATTTAAGATTTCCTTGG-3′ and reverse 5′-CCAAGGAAATCTTAAATATATTTCTCAGCACTTCTTCAACCTCTTC-3′, and were designed using the QuikChange algorithm (Agilent Technologies). Transfection of RKO cells was performed using Lipofectamine 2000 DNA Transfection Reagent (Invitrogen). The resulting stable clones were expanded under G418 selection (1 mg/mL) and confirmed to encode the wild-type *NAT2* sequence by Sanger sequencing using primers forward 5´-GATCCGGTACCGAGGAGAT-3´ and reverse 5´-TTGCTGCCAGATCCTCTTCT-3´.

### RT-qPCR analysis

Direct cDNA extractions were performed from cell lysates using the TaqMan Gene Expression Cells-to-C_T_ Kit (Thermo Fisher Scientific). Overexpression of NAT2*5, NAT2*7 and NAT2*14 transcripts was detected by RT-qPCR using TaqMan probes against *NAT2* (Hs04194721_s1) and the reference gene *ACTB* (Hs01060665_g1). Clones with similar levels of *NAT2* expression were chosen for downstream analyses.

### Western blot

Pellets from 2 × 10^6^ cells were lysed by addition of RIPA buffer (Thermo Scientific). Protein concentration was determined by the Novex BCA assay (Life Technologies) and 50 µg of sample were loaded and separated in a 4–12% SDS-PAGE gel (Life Technologies) at 180 V for 1 h. Sample transfer was performed at 20 V for 8 min using nitrocellulose iBlot Transfer Stacks (Thermo Scientific), which was later blocked with 5% BSA and probed overnight at 4 °C with rabbit Myc-tag (71D10, Cell Signaling Technology) and mouse β-actin (47,778, Santa Cruz) primary antibodies. After incubation with rabbit and mouse secondary antibodies (31,460 and 31,430, Thermo Scientific), proteins were visualized by addition of SuperSignal West Femto Chemiluminescent Substrate (Thermo Scientific) in an ImageQuant LAS 4000 imaging system (GE Healthcare Life Sciences). Immunoblots were quantified in ImageJ and protein expression was quantified relative to β-actin loading control. Unprocessed immunoblot scans are provided in Supplementary Fig. 4.

### Determination of cell growth rate

Cells were plated in six replicate wells of a 96-well plate at a density of 3000 cells per well. After 24 h, all wells were imaged in real time using the IncuCyte ZOOM live cell analysis system (Essen BioScience) and cell confluence was determined every 12 h for a period of 7 days.

### Quantification of NAT2 catalytic activity towards APA

Cells were plated at a density of 18,000 cells per well in a 96-well plate. The next day, different concentrations of the substrate 6-(4-aminophenyl)-N-(3,4,5-trimethoxyphenyl)pyrazin-2-amine (APA) were added to the cells and the reaction was quenched after 30 min by addition of 99.8% MeOH. N-acetyl APA (NAPA) formation was detected by LC–MS/MS on a XEVO TQ (Waters) instrument couples to an Acquity UPLC (Waters) using a HSS T3 column (1.7 µm, 2 × 50 mm). The mobile phases consisted of (A) 0.05% heptafluorobutyric acid and propionic acid, and (B) 0.05% heptafluorobutyric acid and propionic acid in acetonitrile. The *m*/*z* transitions 353 > 323 and 395 > 143 were monitored for APA and NAPA respectively.

### Cell viability assay

Cells were seeded at a density of 8000 cells per well in a 96-well plate. The next day, medium containing different concentrations of APA was added. After 72 h, cell viability was determined in an MTT assay by addition of resazurin (Sigma-Aldrich) followed by fluorescence readouts at 590 nm in a Victor^2^ 1420 microplate reader (Perkin Elmer Wallac).

### NAT2 haplotype resolution by multiplexed single molecule real-time (SMRT) sequencing

Genomic DNA was isolated from the human cancer cell lines RKO, DLD-1, VaCo-703 and HepG2 using the NucleoSpin Kit (Macherey–Nagel) according to the manufacturer’s protocol. The exon 2 of *NAT2* was amplified by PCR with the forward primer 5′-CACACGAGG AAATCAAATGCTA-3′ and the reverse primer 5′-CACTGAGGCCAGATCTCCTATC-3′. The reaction was performed with the following concentrations of the reagents: 1 × Phusion HF Buffer (Thermo Fisher Scientific), 200 µM dNTPs, 0.5 μM forward and reverse primers, 0.02 U/μL Phusion Hot Start II High-Fidelity DNA Polymerase (Thermo Fisher Scientific) and 20 ng of genomic DNA. The PCR was performed in a 2720 Thermal Cycler (Applied Biosystems) following the touch-down protocol with 1 min of initial denaturation at 98 °C, 3 cycles of 98 °C for 30 s, 70 °C for 30 s, 72 °C for 2 min, 3 cycles of 98 °C for 30 s, 68 °C for 30 s, 72 °C for 2 min, 3 cycles of 98 °C for 30 s, 65 °C for 30 s, 72 °C for 2 min, 25 cycles of 98 °C for 30 s, 64 °C for 30 s, 72 °C for 2 min and 10 min of final elongation at 72 °C. Unprocessed agarose gel images are provided in Supplementary Fig. 4. To confirm the amplification of the desired *NAT2* region, Sanger sequencing reactions were prepared containing 20 ng of purified PCR product and 0.1 μM of the corresponding primer. Samples were analyzed in an ABI3730XL DNA Analyzer (ThermoFisher). To perform multiplex SMRT sequencing, the same primer pairs were tagged with an unique barcode sequence of 15 bp (Supplementary Table 2) and a padding sequence of 5 bp. To ensure equimolar representation of all samples, PCR products were pooled by two strategies: (a) quantification by Qubit (LifeTechnologies) fluorometric measurements and (b) quantification of serially diluted PCR products (10,000×, 50,000× and 200,000×) by RT-qPCR analysis. All reactions were prepared with SYBR-Green PCR Master Mix (Applied Biosystems) and performed in a StepOnePlus Real-Time PCR System instrument (Thermo Fisher Scientific). For SMRT sequencing, 2 µg of DNA was provided for each sample. Library preparation and sequencing was performed in a Pacific Biosciences RSII instrument at the National Genomics Infrastructure (NGI) of SciLifeLab, Uppsala, using the PacBio RS II system (Pacific Biosciences). SMRT cells were prepared on individual amplicon pools and sequenced on 1 SMRT cell each according to the manufacturer’s instructions using the P6-C4 chemistry. The obtained sequencing reads were aligned to the human reference genome assembly (GRCh38) with the in-house ComPass aligner (Stoimenov et al., manuscript under preparation). The sequencing coverage of each allele was estimated after classification of the read sequence into one of the identified classes and counting events at each polymorphic site. The final haplotype calling was performed by visual inspection of the sequencing data in the Integrate Genomics Viewer 3.0 (IGV, Broad Institute).

### Estimation of NAT2 allelic frequencies in the 1000 Genomes Project dataset

The full chromosome 8 dataset for 2054 individuals from 1000 Genomes Project Phase 3 (Release 20,150,220) was downloaded from [ftp://ftp.1000genomes.ebi.ac.uk/vol1/ftp/release/20130502/]. The genomic variation in the coding part of *NAT2* was extracted in a form of phased alleles for each individual. The *NAT2* allele definitions were taken from^12^ and matched to the variation at the rs200893121, rs72466456, rs45477599, rs149283608, rs1801279, rs1041983, rs1801280, rs45532639, rs146405047, rs12720065, rs139351995, rs1799929, rs72554617, rs79050330, rs1799930, rs45618543, rs56387565, rs138707146, rs45518335, rs55700793, rs1208, rs56393504 and rs1799931 positions in the alleles from the 1000 Genomes Project to identify the allele group and the specific *NAT2* alleles present in each individual.

### Whole genome sequencing of a Swedish cohort of colorectal cancers and patient-matched normal samples

Tumor and normal tissue samples were initially sectioned from a cohort of 173 stage I-III colorectal cancer patients. During this process, two patients were removed from the study cohort due to mismatches found between their tumor and corresponding normal samples. DNA was extracted from each sample in a Freedom EVO robot (Tecan) using magnetic nanoparticles. To discard tumors with microsatellite instability (MSI), PCR-based MSI detection was performed with the MSI Analysis System Version 1.2 (Promega), where the insertion or deletion of the repeating units NR-21, BAT-26, BAT-25, NR-24, MONO-27, Penta D and Penta C was evaluated. Fragments were sequenced in a 96-capillary 3730xl DNA Analyzer (Thermo Fisher Scientific) and MSI panels were analyzed using the GeneMapper software (Thermo Fisher Scientific). We discarded 24 MSI colorectal tumors, and proceeded with the analysis of tumor cell content in the remaining 147 samples. We identified 72 patients with tumors containing > 40% of tumor cell content and discarded 8 patients who had previously received chemotherapy or radiotherapy. We additionally removed samples from 7 patients for which not enough DNA was available for downstream analyses. These selection criteria identified 56 patients with tumor and matched normal samples suitable for whole genome sequencing. Sequencing libraries were prepared in duplicates from 100 ng of DNA by using the TruSeq Nano DNA sample preparation kit (Illumina), targeting an insert size of 350 bp. Sequencing was performed in a HiSeqX System (Illumina) with a paired-end 150 bp read length. The library preparation and sequencing were performed at the SNP&SEQ Technology Platform (ScilifeLab, Sweden). Tumor and normal samples were sequenced with a 90 × read depth. The variant bases in positions corresponding to the SNPs rs1801279, rs1041983, rs1801280, rs1799929, rs144176822, rs1799930, rs1208, rs1799931, rs2552, rs4646247, rs971473, rs45547533, rs721398 and rs721399 were called separately for the normal and tumor samples. The *NAT2* alleles were imputed from the genotype data. The LOH was estimated as allelic imbalance in one or more informative positions, where there was an observed ≥ 10% shift of the number of sequencing reads supporting each allele.

### Genotyping of a Finnish colorectal cancer patient cohort by SNP array

We derived 1611 cases with colorectal adenocarcinoma from the ongoing Finnish CRC collection (colorectal tissue or blood)^31,32^. The tumor and respective normal DNA samples were genotyped with an Infinium Omni2.5–8 (Illumina Inc.) array at the Estonian Genome Center. The B-Allele Frequencies and Log-R ratios for the polymorphisms rs45529434, rs7832071, rs1801279, rs1041983, rs1801280, rs1799929, rs144176822, rs1799930, rs45607939, rs149460636, rs1208, rs1799931, rs2552, rs4646247, rs971473, rs45547533, rs721398 and rs721399 were extracted with Illumina Genome Studio software. The *NAT2* haplotypes of 1511 patients with informative calls at the allele-classifying positions rs1801279, rs1041983, rs1801280, rs1799929, rs72554617, rs79050330, rs1208 and rs1799931 were imputed from the SNP genotypes. The variation in all genomic positions was used estimate LOH by comparing the data from the normal and tumor sample. We considered the allelic imbalances for LOH if the observed shift in B-allele frequency of the tumor compared to normal was ≥ 0.10 (1 S.D. of the B-allele frequencies of the normal samples).

### Multiplexed high throughput sequencing of colorectal cancer patients samples

DNA isolated from 74 fresh frozen tumor and patient matched normal tissues was obtained from CRC patients with stage II-IV disease. Extraction was performed on a liquid handling workstation Tecan Evo 150 MCA LiHa RoMa as previously described^33^. The second *NAT2* exon of each sample was amplified by PCR following the aforementioned protocol, however the primers contained additional sample-unique barcode sequence to allow amplicon identification. The barcode sequences were those recommended by Pacific Biosciences platform and the HPLC-purified primers were obtained from Sigma-Aldrich (Supplementary Table 4). Barcoded PCR products were pooled equimolarly based on the DNA concentration measurement by Qubit BR fluorometric assay (ThermoFisher). Following purification on a DNA-binding column (GeneJET Gel Extraction Kit), 2 pools of normal samples and 3 pools of tumor samples, each containing 2 µg of DNA were sequenced according to the manufacturers’ instructions using the P6-C4 chemistry in 5 separate SMRT cells using a PacBio RS II system at the National Genomics Infrastructure (NGI) of SciLifeLab, Uppsala.

## Supplementary information

Supplementary Information 1.

Supplementary Information 2.
