# Supplementary material for: Defining eligible patients for allele-selective chemotherapies targeting NAT2 in colorectal cancer
Source: Sci Rep. 2020 Dec 31;10:22436. doi: 10.1038/s41598-020-80288-z (PMC7775439; doi:10.1038/s41598-020-80288-z)
Supplement: Supplementary file 1 — Supplementary Information 1. [file 41598_2020_80288_MOESM1_ESM.docx]

# Defining eligible patients for allele-selective chemotherapies targeting NAT2 in colorectal cancer

Veronica Rendo^1,2^, Snehangshu Kundu^1^, Natallia Rameika^1^, Viktor Ljungström^1^, Richard Svensson^3,4^, Kimmo Palin^5,6^, Lauri Aaltonen^5,6^, Ivaylo Stoimenov^1, #^, and Tobias Sjöblom^1, #, *^

**Affiliations:**

^1^Science for Life Laboratory, Department of Immunology, Genetics and Pathology, Uppsala University, SE-751 85 Uppsala, Sweden

^2^Dana-Farber Cancer Institute, 450 Brookline Avenue, 02215 Boston, USA.

^3^Uppsala Drug Optimization and Pharmaceutical Profiling Facility (UDOPP), SciLifeLab Chemical Biology Consortium Sweden (CBCS), Department of Pharmacy, Uppsala University, 751 23 Uppsala, Sweden

^4^SciLifeLab Drug Discovery and Development Platform, ADME of Therapeutics facility (UDOPP), Department of Pharmacy, Uppsala University, 751 23 Uppsala, Sweden

^5^Applied Tumor Genomics Research Program, Faculty of Medicine, University of Helsinki, Biomedicum Helsinki, PO Box 63 (Haartmaninkatu 8), FI-00014, Helsinki, Finland

^6^Department of Medical and Clinical Genetics, University of Helsinki, Biomedicum Helsinki, PO Box 63 (Haartmaninkatu 8), FI-00014, Helsinki, Finland

#Equally contributing authors

* Correspondence to: tobias.sjoblom@igp.uu.se

**Supplementary Figures**

**
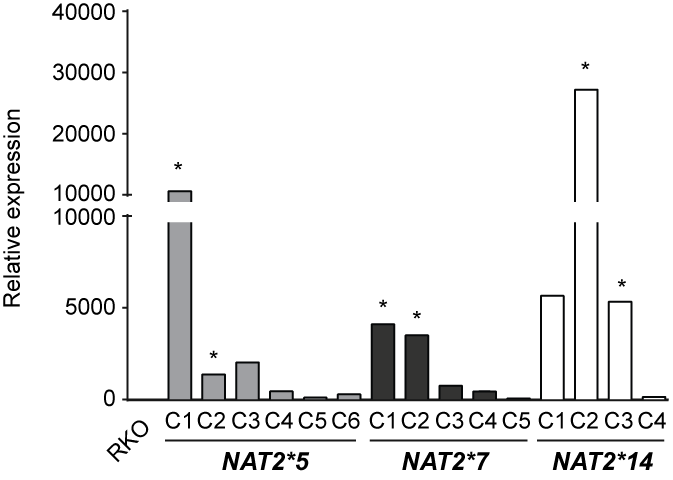
**

**Supplementary Figure 1. Expression analysis of clones encoding *NAT2*5*, **7* and **14* slow acetylator variants after transduction of RKO cells.** The relative expression of each clone was determined by RT-qPCR and normalized towards parental RKO cells. Clones chosen for downstream analyses are shown with an asterisk (*). Data from one representative experiment is shown, where three technical replicates have been included for each clone.

**
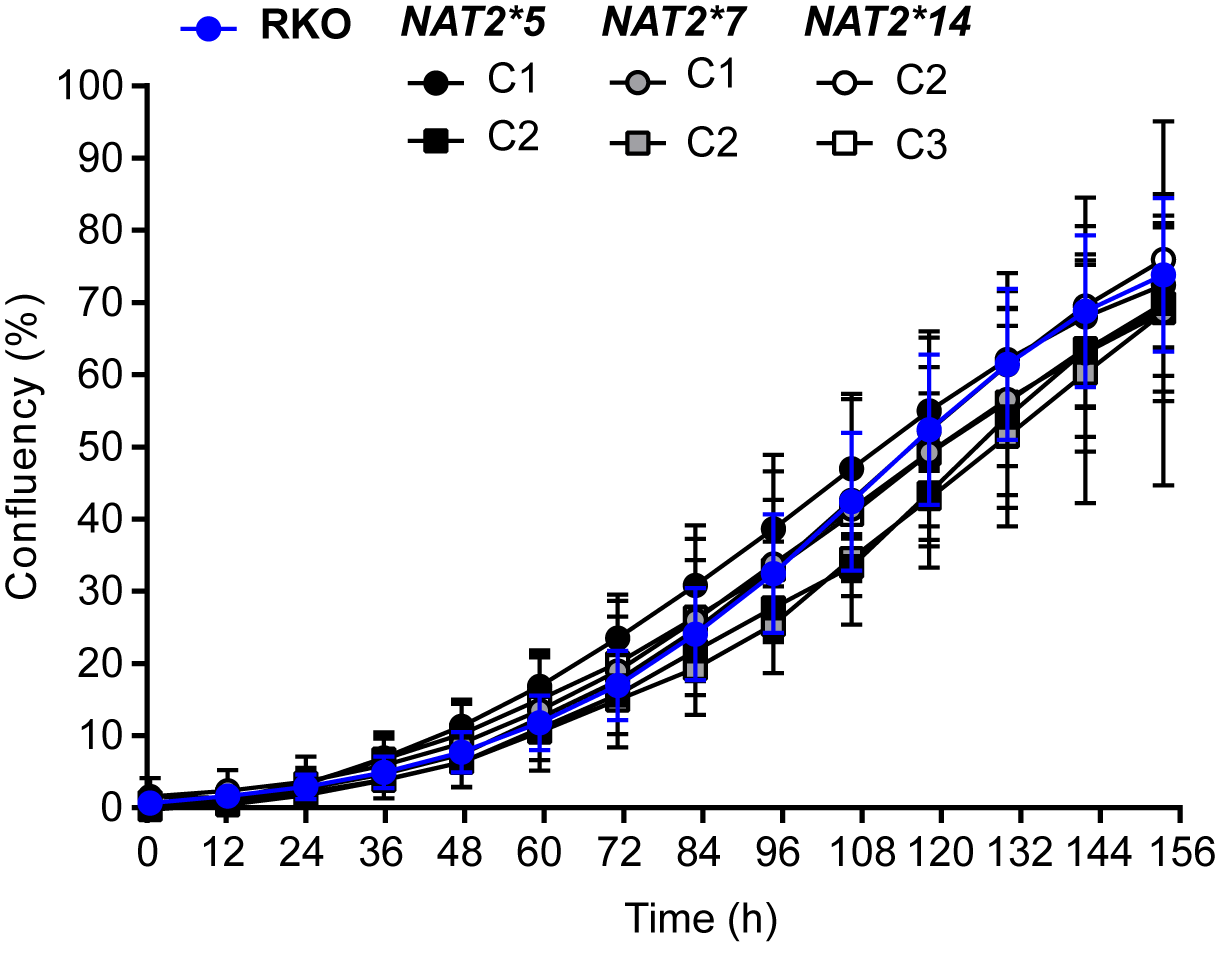
**

**Supplementary Figure 2.** **Tumor cells encoding *NAT2*5*, **7* and **14* enzymatic variants grow at similar rates to parental RKO cells.** The confluency of two clones for each slow acetylator variant was monitored for a period of 156 h. The mean and S.D. of three independent experiments is shown.

**
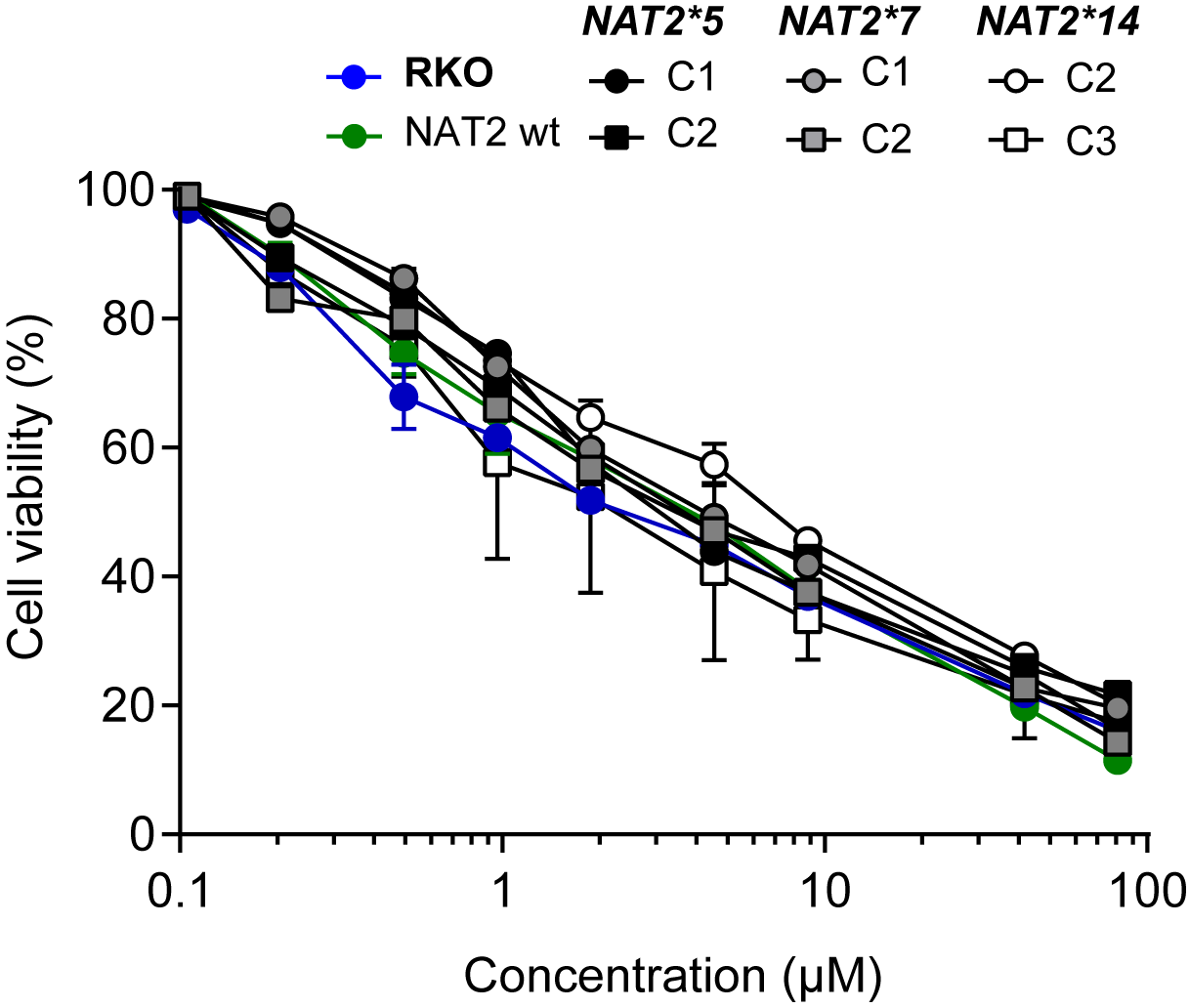
**

**Supplementary Figure 3. Cytotoxicity of 5-fluorouracil is NAT2 independent.** Dose response for fluorouracil (5-FU) in RKO clones expressing the NAT2_5, NAT2_7 and NAT2_14 enzymatic variants. Cell viability was measured by a MTT assay after 72 h. The mean and S.D. of three independent experiments is shown. Data were analyzed using a two-way ANOVA. **, *p* < 0.01 and ****, *p* < 0.0001.


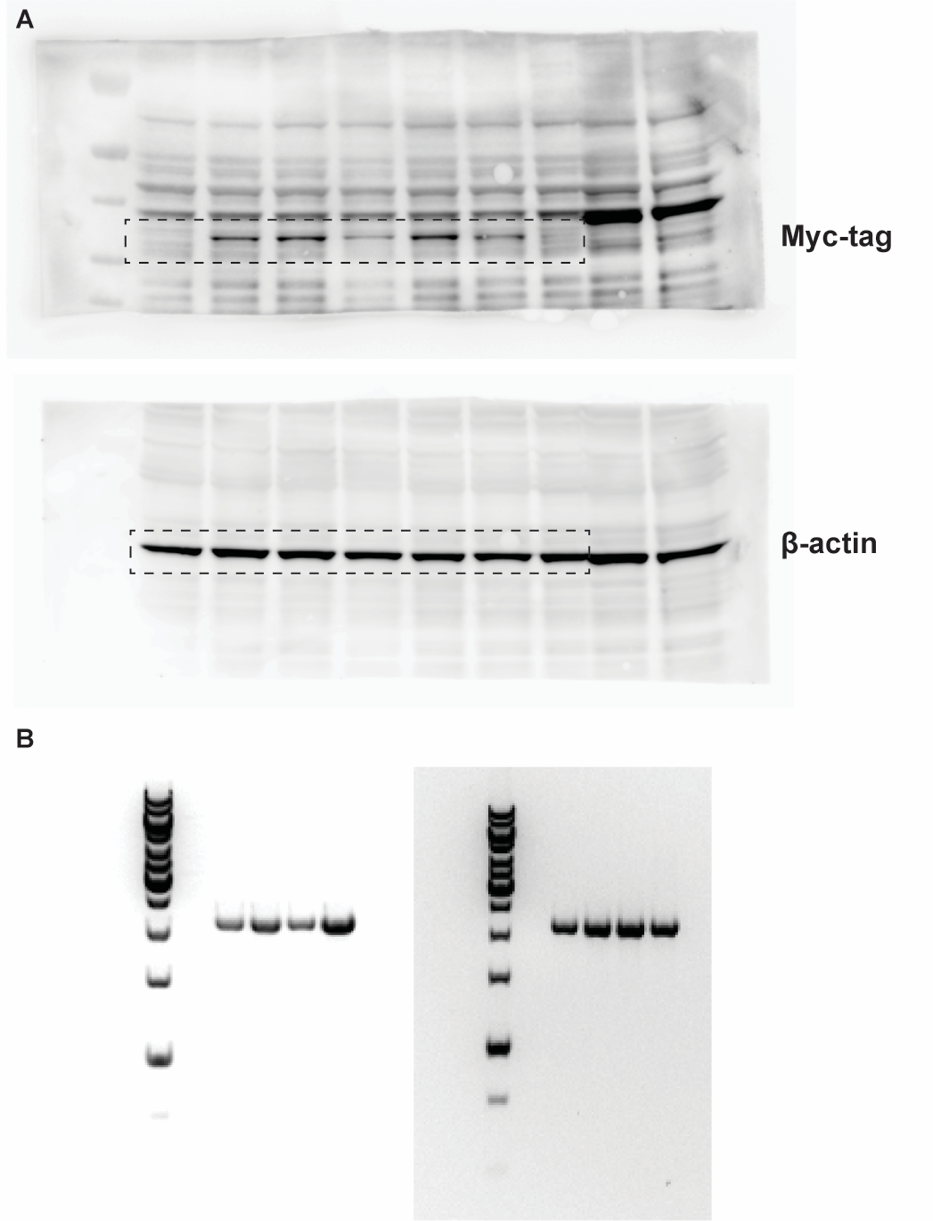


**Supplementary Figure 4.** **Original Western Blot and agarose gel files.**  **(A)** Unprocessed immunoblot scans for Myc-tag and β-actin shown in Figure 2c. The dotted area indicates samples used in this study. **(B)** Unprocessed agarose gel image shown in Figure 5c.

**Supplementary Tables**

**Supplementary Table 1. Estimated number of CRC patients eligible for NAT2 allele-selective treatment.**

| **Haplotype** | **SNP** | **HET frequency*** | **Eligible number of patients**  **(per year)**** |
| --- | --- | --- | --- |
| *NAT2*6* | rs1799930 | 0.367 | Colon: 52,382  Lung: 103,703  Ovarian: 9,639  Head/neck: 16,711 |
| *NAT2*5* | rs1801280 | 0.357 | Colon: 51,002  Lung: 100,970  Ovarian: 9,386  Head/neck: 16,271 |
| *NAT2*7* | rs1799931 | 0.130 | Colon: 18,572  Lung: 36,768  Ovarian: 3,418  Head/neck: 5,925 |
| *NAT2*14* | rs1801279 | 0.050 | Colon: 7,143  Lung: 14,141  Ovarian: 473  Head/neck: 820 |

*Frequencies obtained from the phase 3 of the 1000 Genomes Project [http://www.1000genomes.org]

** The number of eligible patients was calculated using the following LOH frequencies in human cancers: colon (21%); lung (31%); ovarian (22%) and head/neck (20%). The incidence of each cancer type was obtained from the International Agency for Research on Cancer’s 2012 report [http://globocan.iarc.fr] and one independent statistical report for ovarian cancer^34^.

**Supplementary Table 2. Primer barcodes used for multiplexed SMRT sequencing of cancer cell lines.**

| **Barcode number** | **Forward sequence**  **(5’ – 3’)** | **Reverse sequence**  **(5’ – 3’)** |
| --- | --- | --- |
| 1 | GGTAGGCGCTCTGTGTGCAGC | CCATCTCATATGTAGTACTCT |
| 2 | GGTAGTCATGAGTCGACACTA | CCATCGCGATCTATGCACACG |
| 3 | GGTAGTATCTATCGTATACGC | CCATCTGCAGTCGAGATACAT |
| 4 | GGTAGATCACACTGCATCTGA | CCATCGACTCTGCGTCGAGTC |
| 5 | GGTAGACGTACGCTCGTCATA | CCATCTACAGCGACGTCATCG |
| 6 | GGTAGTGTGAGTCAGTACGCG | CCATCGCGCAGACTACGTGTG |
| 7 | GGTAGAGAGACACGATACTCA | CCATCGTCTCTGCGATACAGC |
| 8 | GGTAGCTGCTAGAGTCTACAG | CCATCAGTATGAGATAGCTCG |

**Supplementary Table 3. *NAT2* genotypes and inferred phenotypes of 74 tumor-normal pairs subjected to long read sequencing.** The complete NAT2 coding sequence was amplified and subject to multiplexed SMRT cell sequencing to allow unambiguous haplotyping of tumor and patient-matched normal tissues.

*[Available as a digital file due to size]*

**Supplementary Table 4. Primer barcodes used for multiplexed SMRT sequencing.**

| **Barcode number** | **Forward sequence** | **Reverse sequence** |
| --- | --- | --- |
|  | **(5’ - 3’)** | **(5’ - 3’)** |
| 1 | GGTAGGCGCTCTGTGTGCAGC | CCATCTCATATGTAGTACTCT |
| 2 | GGTAGTCATGAGTCGACACTA | CCATCGCGATCTATGCACACG |
| 3 | GGTAGTATCTATCGTATACGC | CCATCTGCAGTCGAGATACAT |
| 4 | GGTAGATCACACTGCATCTGA | CCATCGACTCTGCGTCGAGTC |
| 5 | GGTAGACGTACGCTCGTCATA | CCATCTACAGCGACGTCATCG |
| 6 | GGTAGTGTGAGTCAGTACGCG | CCATCGCGCAGACTACGTGTG |
| 7 | GGTAGAGAGACACGATACTCA | CCATCGTCTCTGCGATACAGC |
| 8 | GGTAGCTGCTAGAGTCTACAG | CCATCAGTATGAGATAGCTCG |
| 9 | GGTAGAGCACTCGCGTCAGTG | CCATCGCGACGAGTACTCATG |
| 10 | GGTAGTCATGCACGTCTCGCT | CCATCAGTATCACAGTCGCTG |
| 11 | GGTAGAGAGCATCTCTGTACT | CCATCATCATATGATGCGACA |
| 12 | GGTAGCGCATCGACTACGCTA | CCATCAGACGTAGATCACAGC |
| 13 | GGTAGCGTAGCGTGCTATCAC | CCATCCGTGTCATGCTACTCA |
| 14 | GGTAGATGCTGATGACTGCGA | CCATCTGTGAGACTGCATGTC |
| 15 | GGTAGTGCGTGAGCTGTACAT | CCATCGCTCAGTGCGCTACTG |
| 16 | GGTAGCGATCATCTATAGACA | CCATCACTATCGCGCACGCAG |
| 17 | GGTAGCGACGTATCTGACAGT | CCATCTGACACTCTGCACGCG |
| 18 | GGTAGCACGTCACTAGAGCGA | CCATCCAGACGTGACTGATAT |
| 19 | GGTAGTGTCGCAGCTACTAGT | CCATCGCACTGTAGTGATCGT |
| 20 | GGTAGCATACGCTGTGTAGCA | CCATCCAGTGCGAGACAGTAG |
| 21 | GGTAGAGTCGCATGACTGTGT | CCATCAGTAGTGCTACTCGAC |
| 22 | GGTAGCAGTACTGCACGATCG | CCATCATGCGAGATCTGCTCA |
| 23 | GGTAGGTGCTGAGCATCAGAC | CCATCTGAGACATACTGAGTG |
| 24 | GGTAGCACTGATCGATATGCA | CCATCATGTGCACTAGTGTAC |
| 25 | GGTAGTACAGTGTCTGCTGCG | CCATCTCAGCTGACGATGTGA |
| 26 | GGTAGTACAGATAGTGTAGCG | CCATCACTGATGCGCACATGT |
| 27 | GGTAGTCGTAGAGCTCGAGAC | CCATCCTACTCTCAGCAGTGA |
| 28 | GGTAGGAGCTGCGCACTCGAT | CCATCATCTACATCACGACTC |
| 29 | GGTAGGCGATGTCGCTATGTG | CCATCATATAGTACAGCGTCT |
| 30 | GGTAGCGAGAGTCAGCGCATA | CCATCGACACGACTAGATCGC |
| 31 | GGTAGTCACGATGAGCACGTA | CCATCTACGAGTCTGTCATAC |
| 32 | GGTAGGACTGAGATCATGATC | CCATCACTCAGCTACATAGTG |
| 33 | GGTAGACGACATGATACTGCT | CCATCACGTATCATAGTGAGA |
| 34 | GGTAGATACAGCACAGATGTG | CCATCGAGTCGTATCGCTCAT |
| 35 | GGTAGACAGTCGATATCTCTC | CCATCGCGATCACGAGTAGAC |
| 36 | GGTAGGCTCGATCACATGACG | CCATCCTAGACGTACATGTCG |
| 37 | GGTAGGTCGTACACGTGCGAC | CCATCTAGCAGTCACTGTGCG |
| 38 | GGTAGACTCATATCTAGAGTG | CCATCCGTCATGCGATAGCTA |
| 39 | GGTAGACTGATCTGTCGCGCT | CCATCGCGCAGTCGTCTGTAT |
